# Interaction of Hypertension and Insulin Resistance Exacerbates the Occurrence of Diabetes Mellitus in Healthy Individuals

**DOI:** 10.1155/2022/9289812

**Published:** 2022-04-19

**Authors:** Baomin Wang, Yumei Yang, Xiaomu Li

**Affiliations:** Department of Endocrinology and Metabolism, Zhongshan Hospital, Fudan University, Shanghai 200032, China

## Abstract

**Objective:**

Hypertension and type 2 diabetes are common complications. Patients with hypertension often show insulin resistance. The purpose of this study was to investigate the correlations between different blood pressure levels and different degrees of insulin resistance, as well as their interactions, with newly diagnosed diabetes mellitus.

**Methods:**

We conducted a retrospective study on 1251 adult medical examiners who were examined in the Physical Examination Center of Zhongshan Hospital, Fudan University (Shanghai, China) during 2015. All human subjects had no history of diabetes. General clinical data, including blood pressure, fasting glucose and 2-h post-load glucose levels, and lipid profiles, were collected. HOMA-IR was separately calculated. Statistical analyses were carried out by using SPSS software (version 13.0).

**Results:**

In 1251 physical examination subjects, a total of 166 cases of newly diagnosed diabetes were detected, with a total detection rate of 13.3%. The rates of newly diagnosed diabetes in the normal blood pressure group, high-normal blood pressure group, and hypertension group were 4.9%, 10.6%, and 19.0%, respectively. Compared with the normal blood pressure group, the proportion of newly diagnosed diabetes in the hypertension group was significantly increased [OR: 2.956, 95% CI 1.736-5.032, *P* < 0.001]. According to the stratification of HOMA-IR level, with the first quartile group (HOMA-IR<1.21) as a reference, the risk of newly diagnosed diabetes in the fourth quartile group (HOMA-IR ≥2.68) was significantly increased. After adjusting for gender and age, for every unit increase in HOMA-IR, the risk of developing newly diagnosed diabetes increased 9.67 times [OR: 9.670, 95% CI 5.086-18.384, *P* < 0.001]. When hypertension was combined with insulin resistance (HOMA-IR ≥2.68), the risk of newly diagnosed diabetes was 38.32 times compared with the control group [OR: 38.315, 95% CI 9.227-159.108, *P* < 0.001].

**Conclusions:**

Elevated blood pressure levels and insulin resistance levels were associated with the risk of newly diagnosed diabetes. Hypertension was an independent risk factor for newly diagnosed diabetes, and the combination of hypertension with insulin resistance further increased the risk of newly diagnosed diabetes.

## 1. Introduction

The prevalence of diabetes is increasing. It has become the eighth leading cause of death in the world [1]. According to statistics, there were approximately 451 million adult diabetic patients worldwide in 2017, and this number is expected to increase to 693 million by 2045 [2]. Hypertension and type 2 diabetes are common complications. Compared with individuals with normal blood pressure (BP), hypertension has a higher risk of developing diabetes. Previous prospective cohort studies showed that increased BP is an important independent predictor of newly diagnosed diabetes [3, 4]. About 50% of diabetic patients suffer from hypertension at the same time, and the risk of cardiovascular disease in diabetic patients with hypertension is 4 times that of diabetic patients with normal BP [5]. Obesity, inflammation, oxidative stress, and insulin resistance are considered common pathways for hypertension and diabetes [6]. Patients with hypertension often show insulin resistance [7, 8], and hypertension and insulin resistance are independent predictors of newly diagnosed diabetes mellitus. However, no studies have evaluated the predictive value of newly diagnosed diabetes in the presence of both hypertension and insulin resistance. Insulin resistance and high blood pressure interaction may form a vicious circle and jointly promote the occurrence of diabetes. This study aimed to explore the predictive value of different BP levels for the risk of newly diagnosed diabetes under different insulin resistance states, so that we can focus on screening high-risk groups that require key intervention.

## 2. Methods

### 2.1. Ethics, Content, and Permission

All procedures were carried out in compliance with the Helsinki Declaration. The present study was approved by Zhongshan Hospital ethics committee, Fudan University, China. All the participants signed the informed consent.

### 2.2. Study Population

This study was a retrospective clinical controlled study. Participants were continuously recruited from the Physical Examination Center of Zhongshan Hospital, Fudan University, Shanghai, China, in 2015. This study included healthy adults who had no known diabetes before. The exclusion criteria include (1) the presence of diabetes or other serious chronic diseases, such as cardiovascular and cerebrovascular diseases, kidney disease; (2) receiving hypoglycemic therapy; (3) severe infection and acute trauma; (4) mental illness, stress; and (5) failure to sign an informed consent form. Finally, this cross-sectional study included 1251 (462 males and 789 females) subjects aged 63.2 ± 10.2 years. We used the 1999 World Health Organization standards to diagnose diabetic patients. Diabetes is defined as fasting blood glucose ≥7.0 mmol/L or 2 h post-load glucose ≥11.1 mmol/L.

### 2.3. General Clinical Information Collection and Laboratory Examination

Collecting the medical records of selected participants, including general conditions, ergonomic indexes, systolic blood pressure (SBP), diastolic blood pressure (DBP), fasting and postprandial blood glucose, blood lipid, and abdominal circumference. Anthropometric indicators were collected by professionally trained nurses. BP measurement: let the subject rest in a quiet environment for 5-10 minutes before measurement. Instruct the examinee to take a seat, take off his sleeves, and keep the elbow at the same height as the right atrium. Return the reading of mercury sphygmomanometer to zero. Wrap the sphygmomanometer cuff close to the skin around the upper arm, and its lower edge is 2-3 cm above the cubital fossa. The tightness is appropriate for BP measurement. Repeat the measurement at an interval of 1-2 minutes, take the average of the two readings, and record. Glucose oxidase method was used to determine plasma glucose level. Serum insulin level was measured by radioimmunoassay. The blood lipid profile was determined by standard enzymatic test, and the low-density lipoprotein cholesterol (LDL-C) concentration was calculated by Fried Ewald equation. Calculate the homeostasis model insulin resistance index (HOMA-IR): fasting blood glucose (mmol/L) × fasting insulin (mU/L)/22.5. BP level stratification: normal BP (SBP<130 mmHg and DBP<80 mmHg), normal high BP (SBP 130-139 mmHg and/or DBP 80-89 mmHg), and hypertension (SBP ≥140 mmHg and DBP ≥90 mmHg) or patients who have been treated [9]. Diagnostic criteria for diabetes mellitus were diabetes diagnosis standard of WHO in 1999 [10]. Oral glucose tolerance test (OGTT) method: 1. Starting from 7 to 9 a.m., the participant orally took 75 g of anhydrous glucose powder dissolved in 300 ml of water after fasting (8-10 h), such as 82.5 g of glucose in 1 molecular water. The sugar water was consumed within 5 minutes. 2. The time was counted from the first mouthful of oral sugar water. Blood samples were taken from the forearm before and 2 h after taking sugar to measure venous blood glucose.

### 2.4. Statistical Analysis

SPSS 17.0 software was used for statistical analysis. Measurement data conforming to normal distribution were expressed as mean ± standard deviation. T-test was used for the comparison between the two groups and ANOVA was used for the comparison between the three groups. The data that do not conform to the normal distribution were represented by the median (interquartile interval) and rank sum test is performed. *P* < 0.05 indicated the difference is statistically significant. Logistic regression analysis was used to screen the risk factors of newly diagnosed diabetes mellitus. GraphPad prism 8 and Origin 2021 were used for mapping.

## 3. Results

### 3.1. General Clinical Characteristics of the Study Population

The characteristics of the participants are shown in Table 1. A total of 1251 subjects were included in this study, including 462 male patients and 789 female patients, with an average age of 63.2 ± 10.2 years. According to the BP level, the subjects were divided into three groups: normal BP, normal high BP, and hypertension. The basic information and metabolic indexes of each group are shown in Table 1. As BP increased, age, fasting blood glucose, postprandial blood glucose, triglyceride, and abdominal circumference all showed an upward trend. Among 1251 physical examination patients without a history of diabetes, 166 cases were detected as diabetes mellitus, and the proportion of diabetic patients was 13.3%. Among them, 18 cases, 25 cases, and 123 cases of diabetes were detected in the normotensive group, the normal high BP group, and the hypertension group, respectively. The proportions of DM patients were 4.9%, 10.6%, and 19%, respectively. As BP levels increased, the proportion of newly diagnosed diabetes and HOMA-IR both show an upward trend. There was no significant difference in plasma total cholesterol and low-density lipoprotein cholesterol among the groups.

LDL-C: low-density lipoprotein cholesterol; HDL-C: high-density lipoprotein cholesterol; HOMA-IR: homeostasis model assessment of insulin resistance.

### 3.2. The Relationship between Different BP and HOMA-IR Levels and Newly Diagnosed Diabetes

In the joint analyses, we computed the odds ratio (OR) for new-onset diabetes in a multiple logistic regression model in which combinations of HOMA-IR and BP. The results are shown in Table 2. Compared with patients in the normal BP group (BP <130/80 mmHg), there was no statistically significant increase in the proportion of newly diagnosed diabetes in the high-normal BP group (BP 130-139/80-89 mmHg) (*P* = 0.118); however, the proportion of newly diagnosed diabetes in the hypertension group increased significantly [OR: 2.956, 95% CI(1.736-5.032),*P* < 0.001]. After adjusting for confounding factors such as gender, age, BMI, and triglycerides, the difference was still statistically significant [OR: 2.230, 95% CI(1.283-3.876), *P* = 0.004]. According to the HOMA-IR level stratification, with HOMA-IR<1.21 as a reference, the risk of newly diagnosed diabetes increased significantly when HOMA-IR ≥2.68. After adjusting for gender and age, HOMA-IR increased the risk of newly diagnosed diabetes by 9.67 times [OR: 9.670, 95% CI (5.086-18.384), *P* < 0.001] per unit increase.

Model 1: unadjusted. Model 2: adjusted for age and gender. Model 3: adjusted for age, gender, BMI, and triglycerides.

### 3.3. The Effect of BP Combined with Insulin Resistance on Newly Diagnosed Diabetes

**Figure 1:**
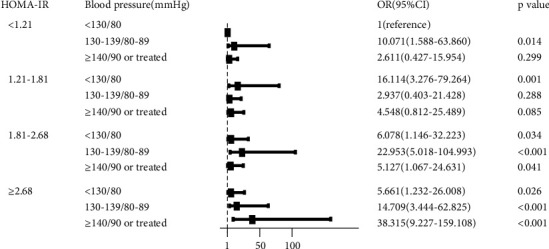
The association between HOMA-IR, BP, and newly diagnosed type 2 diabetes.

**Figure 2:**
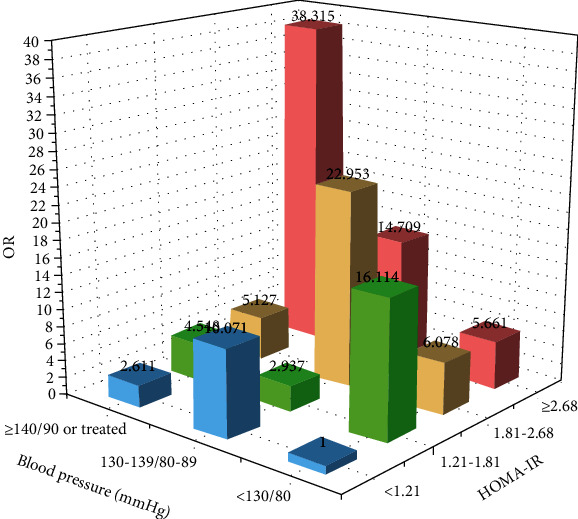
The relationship between HOMA-IR, BP, and new-onset diabetes.

To further explore the relationship between different BP levels and insulin resistance levels with newly diagnosed diabetes, we established hierarchical logistic regression models using stratification by BP level and HOMA-IR level to stratify. Participants were reclassified into 12 subgroups (Figure 1, Figure 2). Taking HOMA-IR<1.21 and BP<130/80 mmHg as a reference, individuals with higher BP levels and insulin resistance levels tended to have higher odds of newly diagnosed diabetes. When combined with HOMA-IR ≥2.68 and hypertension, the risk of newly diagnosed diabetes was 38.32 times than that of the control group [OR: 38.315, 95% CI (9.227-159.108), *P* < 0.001].

## 4. Discussion

The present cross-sectional study found a significantly increased risk of newly diagnosed diabetes among individuals with hypertension compared with normotensive individuals, and furthermore, when BP was increased in combination with insulin resistance, the risk of newly diagnosed diabetes was further increased. The interactive effects between increased BP levels and an elevated HOMA-IR suggest that the accumulation of insulin resistance might interact with and exaggerate the risk of diabetes in hypertensive patients.

Our study found that the prevalence of newly diagnosed diabetes in the hypertensive population was 19.0%, representing a 2-fold as high as the normotensive group. The prevalence of newly diagnosed diabetes in the high-normal BP group was 10.6%, which was 64% higher compared with normotensive subjects. There are conflicting results of previous studies on the relationship between elevated BP and the risk of newly diagnosed diabetes. Most studies concluded that patients with hypertension were at increased risk of newly diagnosed diabetes regardless of antihypertensive treatment. In the Losartan Intervention for Endpoint Reduction in Hypertension Study (LIFE) in which subjects were randomized to antihypertensive treatment based on losartan or atenolol, the results showed that during a median follow-up period of 4.8 years, 562 (7.0%) of 7998 hypertensive patients without a history of diabetes mellitus developed diabetes, and that hypertensive patients had an 18% increased risk of newly diagnosed diabetes for every 10 mmHg increase in systolic blood pressure (SBP) [11]. Verdecchia et al. followed up patients with initially untreated hypertensive for up to 16 years and of the 743 non-diabetic patients, 43 (5.8%) developed diabetes. Logistic regression analysis found that the fasting blood glucose (*P* < 0.001) and the use of diuretics to lower BP during the follow-up period (*P* = 0.004) were independent predictors of newly diagnosed diabetes. Poor BP control can increase the risk of newly diagnosed diabetes by two times [12]. The results of a large prospective cohort study in the United Kingdom showed that each 20 mmHg increase in SBP can increase the risk of newly diagnosed diabetes by 58% (HR =1.58, 95% CI: 1.56-1.59), while each 10 mmHg increase in DBP can increase the risk of newly diagnosed diabetes by 52% (HR =1.52, 95% CI: 1.51-1.54) [13]. Connor A Emdin et al. conducted a meta-analysis of 30 previous prospective cohort studies, and the results showed that for every 20 mmHg increase in SBP, the risk of newly diagnosed diabetes would increase by 77% [13]. Similar conclusions were reached from a large prospective cohort study based on Asian populations, in which Nam h Cho et al. followed 8359 Korean adult individuals without a history of diabetes for up to 10 years, and divided them into 4 groups according to their baseline BP levels: normotensive, prehypertensive, stage 1 hypertension, and stage 2 hypertension, a total of 1195 subjects developed diabetes (14.3%). After adjustment for multiple confounding factors including fasting glucose and two-hour postprandial glucose, individuals with prehypertension, stage 1 hypertension, and stage 2 hypertension had 23%, 26%, and 60% increased risk of newly diagnosed diabetes, respectively, compared with normotensive individuals [14]. However, in a previous prospective cohort study of the risk of incident diabetes among 7097 men, no association was observed between baseline BP and the risk of incident diabetes after correction for clinical and anthropometric measures [15]. Won young Lee et al. followed 14054 Korean nondiabetic adult individuals for an average of 5 years and divided the subjects into three groups by baseline BP: normotensive (<120/80 mmHg), prehypertensive (120/80 mmHg ≤ BP<140/90 mmHg), and hypertensive (≥ 140/90 mmHg). The proportions of newly diagnosed diabetes in the three groups were 0.9%, 1.9%, and 4.0%, respectively. The risk of newly diagnosed diabetes in the hypertensive group was 3.41-fold higher than that in the normotensive group (*P* < 0.0001), but after adjustment for baseline BMI and fasting plasma glucose, there was no significant correlation between BP and newly diagnosed diabetes (*P* = 0.83) [16]. These differences may be due to the limited effectiveness of individual studies and the inability to reliably measure the risk association between BP and newly diagnosed diabetes. Although BP was continuously correlated with the risk of newly diagnosed diabetes, analysis showed that the association between the two was strongest when BP was normal to moderately elevated, and this association tended to be attenuated when BP was above 150 mmHg [13]. Our research also suggests that hypertension is significantly related to the risk of newly diagnosed diabetes, and that maintaining normal BP is an important part of preventing diabetes.

Our study found that there was an interaction between hypertension and insulin resistance in influencing the risk of newly diagnosed diabetes. This result was independent of other traditional metabolic syndrome components such as BMI and plasma triglyceride levels. Insulin resistance is the most important common hub between diabetes and hypertension. Hypertension and insulin resistance were closely related and mutually causal. T Pollare et al. proposed in 1990 that insulin resistance was one of the features of essential hypertension, and that this feature was independent of obesity [8]. E Ferrannini et al. found that in patients with essential hypertension of normal weight and normal glucose tolerance, insulin-induced glucose uptake in peripheral tissues was significantly reduced, that is, patients with essential hypertension were in a state of insulin resistance [17]. Insulin resistance can lead to abnormal vascular function, vascular stiffness, hyperplasia, fibrosis, and remodeling [5]. Insulin resistance specifically manifests as hyperglycemia and compensatory hyperinsulinemia [18]. Hyperglycemia shifts the intracellular fluid outside the cell, leading to an increase in blood volume [19]. Hyperinsulinemia may induce renal sodium retention, renin-angiotensin system activation, enhanced sympathetic nervous system activity, endothelial cell dysfunction, and increased peripheral and renal vascular resistance, contributing to the development of hypertension [20, 21]. Insulin resistance and hypertension interact to create a vicious cycle that together promote the development of diabetes. When the patient has obvious insulin resistance, hypertension can aggravate abnormal glucose metabolism. This study cannot conclude that HOMA-IR is a risk factor for diabetes. The relationship between HOMA-IR and newly diagnosed diabetes is not linear. Previous studies also suggested that HOMA-IR was not linearly related to the diabetes onset [22–24]. Blood glucose level was related to other metabolic indicators. In patients with BP <130/80, HOMA-IR 1.21-1.81 are the highest in risk but those who are higher HOMA-IR are less than this group. It indicated that under normal blood pressure, patients had relatively complete compensatory function. There were other risk factors that ultimately affect the occurrence diabetes, not only HOMA-IR. Therefore, in this group, HOMA-IR was not the risk of diabetes. Moreover, our study population came from routine physical examination population rather than the natural cohort. The average age of the subjects was 63.2 ± 10.2 years old, which is equivalent to middle-aged and elderly adult population. Participants' metabolic states were more complex than the natural population, so the linear relationship cannot be obtained. When patients had significant insulin resistance (HOMA-IR ≥2.68), hypertension had a linear correlation with diabetes mellitus. Hypertension can further predict the risk of diabetes in patients with insulin resistance. Similarly, when the patient was in an obvious state of hypertension (BP ≥140/90), the higher the HOMA-IR was, the higher the risk of getting diabetes. Under such conditions, HOMA-IR was linearly associated with diabetes mellitus. Hypertension interacted with insulin resistance to influence the development of diabetes. This was the reason why we divided the population into 12 components. We hoped to have a clear and intuitive observation of how the two parameters affected the newly diagnosed diabetes. It showed that diabetes was exacerbated by the interactive effects of hypertension and hyperinsulinemia. Our research suggests that patients with both hypertension and insulin resistance have a significantly exponentially higher risk of newly diagnosed diabetes, and they are particularly high-risk groups requiring focused intervention.

A major limitation of the present study was the retrospective cross-sectional clinical study design and cannot establish a causal relationship between hypertension and insulin resistance and newly diagnosed diabetes. In addition, the study subjects were patients undergoing physical examinations in hospitals, and there was a lack of detailed antihypertensive medication records. To further validate our conclusions, a prospective population-based cohort study is needed.

## 5. Conclusion

In summary, our study identified hypertension as a significant risk factor for newly diagnosed diabetes, and the risk of newly diagnosed diabetes in hypertensive patients with insulin resistance was further significantly increased. Patients with hypertension and insulin resistance are a high-risk group for newly diagnosed diabetes, and intensive diabetes prevention and screening measures are warranted in this population.

## Figures and Tables

**Table 1:** Baseline characteristics of study participants [−*x* ± *s* or median (IQR) or *n*].

Characteristics	Total (*n* =1251)	Blood pressure classification(mmHg)			*P* value
		<130/80 (*n* =368)	130-139/80-89 (*n* =236)	≥140/90 or treated (*n* =647)	
Age (y)	63.2 ± 10.2	59.3 ± 8.9	61.1 ± 9.6	66.1 ± 10.3	<0.001
Gender (male/female)	462/789	112/256	81/155	269/378	0.001
Systolic blood pressure (mmHg)	135.8 ± 18.8	116.9 ± 8.6	131.7 ± 6.0	148.0 ± 16.4	<0.001
Diastolic blood pressure (mmHg)	76.9 ± 11.0	69.0 ± 6.4	77.4 ± 7.7	81.3 ± 11.6	<0.001
Fasting blood glucose (mmol/L)	5.3 ± 1.1	5.0 ± 0.8	5.3 ± 0.9	5.5 ± 1.2	<0.001
Postprandial blood glucose (mmol/L)	7.6 ± 3.4	6.4 ± 2.6	7.3 ± 3.0	8.4 ± 3.7	<0.001
Total cholesterol (mmol/L)	5.1 ± 0.9	5.1 ± 0.9	5.1 ± 0.9	5.1 ± 1.0	0.789
Triglycerides (mmol/L)	1.7 ± 1.1	1.5 ± 0.8	1.7 ± 1.2	1.8 ± 1.2	<0.001
LDL-C (mmol/L)	3.0 ± 0.8	3.0 ± 0.8	2.9 ± 0.8	3.0 ± 0.8	0.779
HDL-C (mmol/L)	1.4 ± 0.4	1.5 ± 0.4	1.4 ± 0.4	1.4 ± 0.4	0.001
Abdominal circumference (cm)	83.4 ± 9.7	79.7 ± 8.5	82.6 ± 8.5	85.8 ± 10.0	<0.001
Diabetes (yes/no)	166/1085	18/350	25/211	123/524	<0.001
Proportion of diabetic patients (%)	13.3	4.9	10.6	19.0	<0.001
HOMA-IR	1.8 (1.2-2.7)	1.4 (0.9-2.0)	1.9 (1.3-2.6)	2.0 (1.3-3.1)	<0.001

**Table 2:** The odds ratio (OR) of new-onset diabetes with different BP and HOMA-IR levels [OR (95% CI)].

	Model 1 OR (95% CI)	*P*	Model 2 OR (95% CI)	*P*	Model 3 OR (95%)	*P*
Blood pressure (mmHg)						
<130/80	1		1		1	
130-139/80-89	1.680 (0.877-3.218)	0.118	1.541 (0.797-2.979)	0.199	1.473 (0.760-2.856)	0.252
≥140/90 or treated	2.956 (1.736-5.032)	<0.001	2.432 (1.407-4.202)	0.001	2.230 (1.283-3.876)	0.004
HOMA-IR						
<1.21	1		1		1	
1.21-1.81	1.529 (0.730-3.201)	0.260	1.560 (0.737-3.300)	0.245	1.401 (0.658-2.985)	0.382
1.81-2.68	2.846 (1.445-5.606)	<0.002	3.036 (1.535-6.005)	0.001	2.495 (1.231-5.055)	0.011
≥2.68	8.651 (4.585-16.325)	<0.001	9.670 (5.086-18.384)	<0.001	7.049 (3.479-14.285)	<0.001

## Data Availability

The data that support the findings of this study are available from the corresponding author upon reasonable request.

## Abbreviations

HOMA-IR: Insulin resistance index
SBP: Systolic blood pressure
DBP: Diastolic blood pressure
LDL-C: Low-density lipoprotein cholesterol
OGTT: Oral glucose tolerance test
ORs: Odds ratio
LIFE: The Losartan Intervention for Endpoint Reduction in Hypertension Study.
